# Associations between adolescents’ energy drink consumption frequency and several negative health indicators

**DOI:** 10.1186/s12889-023-15055-6

**Published:** 2023-02-06

**Authors:** Maija Puupponen, Jorma Tynjälä, Raili Välimaa, Leena Paakkari

**Affiliations:** Research Centre for Health Promotion, Faculty of Sport and Health Sciences, University of Jyväskylä, Keskussairaalantie 4, 40600 Jyväskylä, FI Finland

**Keywords:** Energy drinks, Adolescent, Health behavior, Relative risk

## Abstract

**Background:**

The purpose of this study was to identify how various negative health indicators are associated with energy drink consumption frequency among 13- and 15-year-old Finnish adolescents.

**Methods:**

Data (*N* = 2429) from the nationally representative international Health Behaviour in School-aged Children study (2018) were analyzed via descriptive analysis and logistic regression analyses, with control for salient covariates. Relative risks (RR) were derived from the adjusted odds ratios.

**Results:**

Even infrequent energy drink consumption was associated with various negative health indicators. Moreover, as compared to non-users, frequent energy drink consumers were more likely to report several health-compromising behaviors: current smoking (RR = 9.85, 95% CI: 5.68–16.02), current snus use (RR = 3.62, 95% CI: 1.80–6.85), cannabis use (RR = 3.42, 95% CI: 1.69–6.52), alcohol consumption (RR = 3.08, 95% CI: 2.49–3.71), problematic social media use (RR = 2.53, 95% CI:1.68–3.72), short sleep (RR = 2.12, 95% CI: 1.69–2.60), skipping breakfast (RR = 1.87, 95% CI: 1.51–2.29), drunkenness (RR = 1.59, 95% CI: 1.11–2.23), inadequate tooth brushing (RR = 1.28, 95% CI: 1.05–1.54). In addition, frequent energy drink consumption was associated with perceived negative health indicators: feelings of insufficient sleep (RR = 1.56, 95% CI: 1.11–2.15), low self-rated health (RR = 1.48, 95% CI: 1.15–1.87), and multiple health complaints (RR = 1.31, 95% CI: 1.07–1.56).

**Conclusions:**

Energy drink consumption, even infrequent, was associated with several negative health indicators, and the reporting of these increased with the frequency of energy drink consumption. The findings support the concerns of health authorities regarding the negative associations between energy drink consumption and health, even among persons as young as 13 years. There is evidence to support specific policy level actions, including restrictions on the sale of energy drinks to adolescents. This measure has been proposed in a Finnish government program, but implementation has yet to occur. Moreover, marketing of these beverages in platforms that are popular among adolescents (e.g., the social media) should be rigorously evaluated, and comprehensive interventions and actions implemented to ensure that adolescents, parents/guardians, and professionals working with adolescents (e.g., in schools) have a good understanding of the links between energy drink consumption and health.

**Supplementary Information:**

The online version contains supplementary material available at 10.1186/s12889-023-15055-6.

## Background

Adolescence is a rapid developmental phase characterized by major physical, psychological, and psychosocial changes [1]. It is accompanied by the initiation of several health behaviors, which often track forward to adulthood [2]. Adolescents’ energy drink consumption has raised concerns among health authorities in different countries and regions. Energy drinks contain high levels of caffeine along with other plant-based stimulants and ingredients [3, 4]. Mostly due to their stimulant content, there have been a number of statements concerning the inappropriateness for adolescents of energy drinks and their constituents in various regions, including Finland [5], Europe [3], and the United States [6]. Moreover, World Health Organization researchers [7] have viewed energy drinks as a significant future risk for public health. In line with these statements, mandatory labeling has been established in EU member states for products with added caffeine content [8]. The labels include a warning that energy drinks are not recommended for sensitive consumer groups such as children.

Despite these statements, energy drink consumption among adolescents is extremely prevalent across Europe [4, 9], and energy drinks are widely available for adolescents worldwide, due to the fact that like most European countries, the United States does not restrict sales to adolescents [10].

The rationale for the recommendations is mainly based on the known adverse physiological effects; however, a growing body of literature has linked energy drink consumption to the use of other harmful substances including alcohol [9, 11–17], cigarettes [9, 11, 13–16, 18], e-cigarettes [16], cannabis, and various other drugs [11, 14].

It is also important to examine the extent to which energy drinks contribute to other unfavorable health behaviors among adolescents, with consequences such as insomnia and obesity [7], various health complaints [9, 13, 19], and mental health problems [15]. Previous studies have found associations between energy drink consumption and late bedtime [17, 19] and short sleep [14, 20]. In addition, consumption of these beverages has been linked to an unhealthy diet [16–18, 20], higher BMI [14], and lower BMI [12]. Moreover, energy drink consumption has been associated with sedentary behaviors such as higher video game use [18] and higher screen time [17, 20, 21]. It has been further linked both to lower physical activity [14, 21] and to higher physical activity [17]. In contrast, Larson et al. [18] found no association between energy drink consumption and physical activity.

Despite the growing research evidence, studies on adolescents have mostly concentrated on dichotomous measures in comparisons involving energy drink consumption. Thus, some studies have compared consumption versus no consumption, without considering the consumption frequency, while others have included frequency comparisons, but have ruled out non-users. There is also inconsistency in the definitions of different consumer groups. For example, “more frequent consumption” has been specified as consumption of energy drinks more than once a month [11], or else four or more times during the previous week [15].

To conclude, only a few studies (e.g. [11, 12, 15, 19, 22]) on adolescents have compared the associations between energy drink consumption and health indicators, while also giving consideration to the consumption frequency. The studies in question all indicate that frequency should be taken into consideration. Furthermore, most of the studies to date have focused only on individual health behaviors, and have not included groups of behaviors within the same study. They have also failed to include certain specific behaviors that have received too little attention in the literature on energy drinks, such as tooth brushing and the use of snus (i.e., smokeless tobacco).

Given that the knowledge on energy drinks in Finland is scarce, and that there are empirical gaps in the literature related to methodologies and specific indicators, there is a clear need to obtain more evidence on energy drink consumption, and its associations with various health indicators in different cultural contexts and countries. Studies on these lines would build knowledge to improve regulations worldwide [7].

By utilizing nationally representative data, we aimed to address the following two objectives. First of all, we aimed to describe the prevalence of various negative health indicators among different groups of energy drink consumers (categorized as *frequent, infrequent*, and *no consumption*) among 13- and 15-year-old adolescents. The indicators included *health-compromising behaviors* (i.e., inadequate tooth brushing, skipping breakfast, low physical activity, short sleep, problematic social media use, current smoking, alcohol consumption, drunkenness, current snus use, and cannabis use), and *perceived unfavorable health indicator*s (i.e., low self-rated health, multiple health complaints, and feelings of insufficient sleep). Secondly, we wished to analyze whether consumption frequency played a role, and whether age and gender moderated the associations identified after control for salient covariates.

## Methods

### Design

Cross-sectional data were collected from Finnish adolescents in 2018 as part of the international Health Behaviour in School-aged Children (HBSC) study, which aims to gain information on adolescents’ health and well-being within their social context [23]. The European Union NUTS classification (Nomenclature of Territorial Units for Statistics) was applied in the stratification of the sampling, which was based on the Finnish school register. The participating schools were selected from the national school register using a cluster sampling method adjusted by province, the type of municipality, and the size of the school (via PPS, i.e., Proportion Probable Size). The school principals gave permission for school classes to participate in the study. Within each school, participating classes were randomly selected. Parents were informed, and some schools required active parental/guardian consent. Informed consent was obtained from all students involved in the study, and participation in the study was voluntary. The survey was carried out as an online questionnaire via Webropol software (Webropol Oy, Helsinki, Finland). The response rate of students within schools which promised to take part in the survey was 54.1%.

### Participants

The nationally representative and anonymous data were obtained from a total of 2429 adolescents (13-year-olds, *n* = 1260; 15-year-olds, *n* = 1169) in 77 schools. The proportion of boys (*n* = 1218) and girls (*n* = 1211) was almost equal.

### Measures

Sociodemographic characteristics included age, self-reported gender, and self-reported material family wealth, which was assessed via Family Affluence Scale III (FASIII) [24].

Energy drink consumption was measured by asking adolescents to respond to “How many times a week do you eat or drink the following…” with energy drinks comprising one item in a 5-item block. The response categories were: *never, less than once a week, once a week, 2–4 days a week, 5–6 days a week, once a day every day,* and *every day more than once.* For the analyses in the present study, responses were grouped into three categories: *frequent consumption* (i.e., weekly), *infrequent consumption* (i.e., less than weekly), and *no consumption*.

Health indicators (i.e., health behaviors and perceived health indicators) and their negative values were measured by the items presented in Table 1. Physical activity was evaluated according to the moderate-to-vigorous activity (MVPA) scale [25]. Sleep length was calculated from the time of going to bed to the time of waking up on school days. Problematic social media use was defined by using the 9-item Social Media Disorder (SMD) scale [26], and the risk level was categorized according to Boer et al. [27]. In the perceived health indicators, self-rated health was measured using a single item [28], and health complaints were measured via the HBSC-SCL symptom checklist [29]. We examined the associations by defining a cut-off point indicating the negative dimension of each health indicator; this was based on the risk levels used in the HBSC international report [23] and on national recommendations (e.g., on tooth brushing), with attention also to the distribution of the response.

**Table 1:** Measures on health indicators and their negative dimension

**Question**	**Indicator range**	**Negative value of the indicator**
***Health-related behaviors***		
**Brushing teeth**: *How often do you brush your teeth?*	1 = More than once a day 2 = Once a day 3 = At least once a week but not daily 4 = Less than once a week 5 = Never	**Inadequate tooth brushing:** < 2 times/day (i.e., all values except 1 = More than once a day)
**Eating breakfast**: *How often do you usually have breakfast (more than a glass of milk or fruit juice) on weekdays?*	1 = I never have breakfast during the week 2 = One day 3 = Two days 4 = Three days 5 = Four days 6 = Five days	**Skipping breakfast:** Eating breakfast 0–2 days/weekdays
**Physical activity**: *Over the past 7 days, on how many days were you physically active for a total of at least 60 min per day?*	0 to 7 days	**Low physical activity**: 0–2 days
**Sleep length**: Difference between bedtime and wake-up time on weekdays	Hours	**Short sleep**: ≤ 7 h / day
**Social media use**: *During the past year, have you…* 9 items on the following criteria: preoccupation, tolerance, withdrawal, persistence, displacement, problem, deception, escape, conflict; for example: *… regularly found that you can’t think of anything else but the moment that you will be able to use social media again?*	1 = No 2 = Yes (Items were coded as 1 = Yes and 0 = No, and a sum score was computed)	**Problematic social media use**: Sum score of ≥ 6
**Smoking**: *How often do you smoke tobacco at present?*	1 = Every day 2 = At least once a week, but not every day 3 = Less than once a week 4 = I do not smoke	**Current smoking:** From every day to less than once a week (i.e., all values except 4 = I don’t smoke)
**Snus use**: *Do you currently use snuff/snus?* (Question was only asked of 15-year-olds)	1 = Every day 2 = Every week, but not daily 3 = Less than once a week 4 = I don’t use snuff/snus	**Current snus use**: From every day to less than once a week (i.e., all values except 4 = I don’t use snuff/snus)
**Alcohol consumption**: *On how many days (if any) have you drunk alcohol?* *… In the last 30 days?*	1 = Never 2 = 1–2 days 3 = 3–5 days 4 = 6–9 days 5 = 10–19 days 6 = 20–29 days 7 = 30 days (or more)	**Alcohol consumption**: All values except 1 = Never
**Drunkenness**: *Have you ever had so much alcohol that you were really drunk?* (Question was only asked of 15-year-olds) *… In your lifetime?*	1 = No, never 2 = Yes, once 3 = Yes, 2–3 times 4 = Yes, 4–10 times 5 = Yes, more than 10 times	**Drunkenness**: Twice or more
**Cannabis use**: *Have you ever taken cannabis (for example marihuana)?* *… In your lifetime?* (Question was only asked of 15-year-olds)	1 = Never 2 = 1–2 days 3 = 3–5 days 4 = 6–9 days 5 = 10–19 days 6 = 20–29 days 7 = 30 days (or more)	**Cannabis use**: All values except 1 = Never
***Perceived health indicators***		
**Self-rated health**: *Would you say your health is…?*	1 = Excellent 2 = Good 3 = Fair 4 = Poor	**Low self-rated health**: Fair or poor
**Health complaints**: *In the last 6 months: how often have you had the following…? headache, stomach-ache, backache, feeling low, irritability or bad temper, feeling nervous, difficulties in getting to sleep, feeling dizzy*	1 = About every day 2 = More than once a week 3 = About every week 4 = About every month 5 = Rarely or never (Items were coded as 1 = More than once a week or about every day and 0 = About every week or less, and a sum score was computed)	**Multiple health complaints:** Sum score of ≥ 2 (i.e., two or more symptoms more than once a week)
**Sleep sufficiency**: *How often do you feel that you have slept sufficiently?*	1 = Every or almost every morning 2 = 3–5 mornings a week 3 = 1–2 mornings a week 4 = Hardly ever	**Feelings of insufficient sleep:** Slept sufficiently hardly ever

### Statistical analysis

Descriptive analysis (including percentages, confidence intervals, and *p*-values) was used to determine the prevalence of negative health indicators by energy drink consumption. This information was stratified by age, and also presented for the total sample. Logistic regression models were used to examine the associations between energy drink consumption frequency and negative health indicators; this was done via a separate model for each health indicator. Models were individually controlled for age, gender, family affluence, and a set of other covariates (health-compromising behaviors and multiple health complaints). This was done on a theoretical basis according to which health-compromising behaviors accumulate and co-occur (e.g. [30]). Pairwise multiple comparisons were performed for each model with the Šidák correction test. To test whether age and gender moderated the associations between energy drink consumption and negative health indicators, the following analyses were conducted: (i) two-way interactions were tested in all models; (ii) for those models showing a statistically significant interaction the adjusted effect for energy drink consumption was estimated; furthermore, (iii) separate models by gender and/or by age were formed (see also the results, and the tables in the Additional files 3 and 4). Relative risks were derived from the adjusted odds ratios to provide a more accurate interpretation of the associations, using the following formula [31]:

$$\mathrm{RR}=\frac{\mathrm{OR}}{\left(1-{\mathrm{P}}_{0}\right)+({\mathrm{P}}_{0}\times \mathrm{OR})}$$

in which P_0_ indicates the prevalence of the outcome of interest (negative health indicator) in the reference group (no energy drink consumption). The analyses were carried out using Stata (version 16) [32]. The analyses were weighted by language group (Finnish and Swedish) and by grade level.

## Results

### Prevalence of negative health indicators among energy drink consumers

Half of the adolescents who frequently consumed energy drinks reported brushing their teeth less than twice a day, and consuming alcohol over the past 30 days (Table 2). Moreover, in the category of frequent energy drink consumers, less than half reported multiple health complaints, two out of five reported skipping breakfast and having short sleep, one in three reported current smoking and low self-rated health, one in four reported feelings of insufficient sleep, while one in five reported low physical activity, with a similar proportion responding in a manner that categorized them as problematic social media users.

**Table 2:** Prevalence of health-compromising behaviors and perceived negative health indicators^a^ by energy drink consumption among 13- and 15-year-olds

	**13-year-olds**				**15-year-olds**				**Total**			
	**Energy drink consumption**				**Energy drink consumption**				**Energy drink consumption**			
	**Frequent**	**Infrequent**	**No consumption**	***P*-value**^**b**^	**Frequent**	**Infrequent**	**No consumption**	***P*-value**^**b**^	**Frequent**	**Infrequent**	**No consumption**	***P*-value**^**b**^
Inadequate tooth brushing	53.6 [46.7–60.4]	42.2 [35.9–48.8]	26.3 [22.4–30.7]	< 0.001	47.9 [41.7–54.1]	35.1 [30.4–40.2]	30.3 [25.4–35.7]	< 0.001	50.3 [45.6–55.0]	38.3 [34.4–42.3]	28.1 [25.0–31.4]	< 0.001
Skipping breakfast	33.9 [28.7–39.6]	20.7 [15.4–27.3]	13.4 [10.7–16.6]	< 0.001	41.1 [35.2–47.2]	21.4 [16.7–27.1]	18.1 [14.5–22.4]	< 0.001	38.1 [33.9–42.4]	21.1 [17.4–25.4]	15.5 [13.2–18.0]	< 0.001
Low physical activity	15.7^c^ [11.3–21.3]	6.8^d^ [3.9–11.6]	10.3^e^ [7.7–13.6]	0.019	25.9^c^ [21.8–30.5]	17.9^d^ [13.6–23.1]	20.4^e^ [16.8–24.5]	< 0.001	21.6 [18.3–25.4]	13.0 [10.1–16.5]	14.7 [12.4–17.4]	< 0.001
Short sleep	39.8 [33.5–46.5]	20.7 [16.4–25.8]	11.1^e^ [8.4–14.4]	< 0.001	38.4 [32.7–44.4]	26.2 [21.3–31.8]	18.4^e^ [14.9–22.4]	< 0.001	39.0 [34.6–43.5]	23.8 [20.4–27.6]	14.3 [12.0–16.9]	< 0.001
Problematic social media use	23.4 [17.2–31.0]	12.4 [10.4–19.9]	6.5 [4.4–9.5]	< 0.001	18.5 [14.5–23.2]	12.8 [9.5–17.2]	5.7 [3.7–8.6]	< 0.001	20.5 [17.0–24.6]	12.6 [9.8–16.2]	6.1 [4.6–8.2]	< 0.001
Current smoking	20.8^c^ [15.8–27.0]	3.2^d^ [1.6–6.3]	0.6^e^ [0.2–1.7]	< 0.001	36.2^c^ [30.1–42.7]	12.3^d^ [8.5–17.4]	3.7^e^ [2.1–6.5]	< 0.001	29.8 [25.2–34.9]	8.3 [5.8–11.6]	1.9 [1.1–3.3]	< 0.001
Alcohol consumption	37.3^c^ [30.4–44.8]	13.0^d^ [8.7–19.0]	6.1^e^ [4.2–8.7]	< 0.001	56.4^c^ [49.0–63.6]	40.3^d^ [33.6–47.5]	20.6^e^ [15.7–26.5]	< 0.001	48.5 [43.0–54.1]	28.2 [23.0–34.1]	12.3 [9.7–15.6]	< 0.001
Drunkenness					45.2 [38.3–52.2]	26.0 [21.8–30.8]	12.4 [9.2–16.5]	< 0.001				
Current snus use					29.8 [25.0–35.0]	10.5 [7.3–15.0]	3.0 [1.9–4.8]	< 0.001				
Cannabis use					25.0 [19.8–31.0]	10.4 [7.1–14.8]	2.9 [1.7–4.9]	< 0.001				
Low self-rated health	37.0^c^ [31.2–43.2]	18.5 [14.1–23.8]	13.3 [10.6–16.7]	< 0.001	25.3^c^ [19.7–31.8]	19.2 [14.7–24.7]	16.8 [13.5–20.7]	0.037	30.2 [25.7–35.2]	18.9 [15.6–22.7]	14.8 [12.7–17.3]	< 0.001
Multiple health complaints	50.4 [42.9–57.9]	33.0 [28.0–38.4]	28.2 [23.5–33.4]	< 0.001	42.5 [37.5–47.7]	39.0 [33.8–44.5]	34.6 [30.0–39.4]	0.080	45.8 [41.3–50.4]	36.4 [32.6–40.4]	31.0 [27.6–34.6]	< 0.001
Feelings of insufficient sleep	26.0 [20.0–33.1]	10.3 [6.9–14.9]	8.5^e^ [6.1–11.6]	< 0.001	25.5 [21.0–30.6]	13.9 [10.6–18.0]	13.0^e^ [10.1–16.6]	< 0.001	25.7 [22.1–29.7]	12.3 [9.8–15.3]	10.5 [8.5–12.8]	< 0.001
*n*	(164–272)	(251–383)	(631–687)		(287–332)	(297–334)	(458–501)		(523–604)	(548–614)	(1089–1186)	

^a^Only the percentage for the negative dimension is presented in the table

^b^*P*-values for the groups of energy drink consumption

^c^Statistically significant difference between the age groups among frequent consumers, Pearson's chi-squared test

^d^Statistically significant difference between the age groups among infrequent consumers, Pearson's chi-squared test

^e^Statistically significant difference between the age groups among non-consumers, Pearson's chi-squared test

Additional items for 15-year-olds regarding substance use indicated that out of the frequent energy drink consumers, less than half reported drunkenness, one in three reported current snus use, and one in four had used cannabis.

In a comparison of age groups (see Additional file 1: Table S5), among both frequent and infrequent consumers, 15-year-olds showed a higher prevalence of low physical activity, current smoking, and alcohol consumption. Among frequent consumers, 13-year-olds showed a higher prevalence of low self-rated health. Among non-consumers, 15-year-olds reported a higher prevalence of low physical activity, short sleep, current smoking, alcohol consumption, and feelings of insufficient sleep. The overall prevalence of health-compromising behaviors and perceived negative health indicators among 13- and 15-year-olds can be found in Additional file 2: Table S6.

### Associations between energy drink consumption frequency and negative health indicators

#### Health-compromising behaviors

The reporting of several health-compromising behaviors (such as short sleep, problematic social media use, current smoking, alcohol consumption, current snus use and cannabis use) increased even among infrequent consumers, as compared to adolescents who did not consume energy drinks (Table 3). This was the case even after control for several salient covariates was applied. By contrast, infrequent energy drink consumers were less likely to report low physical activity than non-consumers.

**Table 3:** Health-compromising behaviors by energy drink consumption: adjusted odds ratios (aOR), 95% confidence intervals (CI), and corresponding relative risks (RR) and 95% confidence intervals (CI)^a^

		**Inadequate tooth brushing**^**b**^	**Skipping breakfast**^**b**^	**Low physical activity**^**c**^	**Short sleep**^**d**^	**Problematic social media use**^**b**^	**Current smoking**^**e**^	**Alcohol consumption**^**f**^	**Drunkenness**^**b**^	**Current snus use**^**b**^	**Cannabis use**^**b**^
Energy drinks											
No consumption		1.00	1.00	1.00	1.00	1.00	1.00	1.00	1.00	1.00	1.00
Infrequent consumption	aOR 95% CI *P*-value	1.22 [0.94–1.58] 0.139	1.24 [0.89–1.72] 0.196	0.70 [0.50–0.99] 0.045	1.62 [1.24–2.12] < 0.001	2.23 [1.40–3.56] 0.001	3.15 [1.56–6.37] 0.002	2.28 [1.63–3.18] < 0.001	1.39 [0.85–2.28] 0.187	2.63 [1.27–5.43] 0.009	2.59 [1.22–5.49] 0.014
	RR 95% CI	1.15	1.20	0.73	1.49	2.07	3.03	1.97	1.33	2.51	2.48
		[0.96–1.36]	[0.91–1.55]	[0.54–0.99]	[1.20–1.83]	[1.37–3.08]	[1.54–5.78]	[1.51–2.51]	[0.87–1.97]	[1.26–4.79]	[1.21–4.86]
Frequent consumption	aOR 95% CI *P*-value	1.44 [1.07–1.94] 0.016	2.22 [1.66–2.99] < 0.001	0.83 [0.55–1.26] 0.373	2.60 [1.91–3.54] < 0.001	2.81 [1.76–4.51] < 0.001	11.89 [6.25–22.59] < 0.001	4.34 [3.14–5.99] < 0.001	1.74 [1.13–2.70] 0.013	3.94 [1.85–8.37] < 0.001	3.68 [1.73–7.80] 0.001
	RR 95% CI	1.28	1.87	0.85	2.12	2.53	9.85	3.08	1.59	3.62	3.42
		[1.05–1.54]	[1.51–2.29]	[0.59–1.21]	[1.69–2.60]	[1.68–3.72]	[5.68–16.02]	[2.49–3.71]	[1.11–2.23]	[1.80–6.85]	[1.69–6.52]
Infrequent vs. Frequent^g^	*P*-value	0.562	0.003	0.803	0.005	0.408	< 0.001	< 0.001	0.642	0.456	0.619

Interaction was found in the models for short sleep and alcohol consumption: adjusted effect presented. The interaction effect is presented in the Additional file 3: Table S7

^a^Relative risks and their confidence intervals were derived from adjusted odds ratios

^b^Models were adjusted for age, gender, family affluence, short sleep, low physical activity, current smoking, alcohol consumption, and multiple health complaints

^c^Model was adjusted for age, gender, family affluence, short sleep, current smoking, alcohol consumption, and multiple health complaints

^d^Model was adjusted for age, gender, family affluence, low physical activity, current smoking, alcohol consumption, and multiple health complaints

^e^Model was adjusted for age, gender, family affluence, short sleep, low physical activity, alcohol consumption, and multiple health complaints

^f^Model was adjusted for age, gender, family affluence, short sleep, low physical activity, current smoking, and multiple health complaints

^g^Tested with pairwise multiple comparisons

Frequent energy drink consumers were more likely than non-consumers to report all listed health-compromising behaviors, except for low physical activity. They were also more likely than infrequent consumers to report skipping breakfast, short sleep, current smoking, and alcohol consumption.

Age moderated the association between energy drink consumption and short sleep, and between energy drink consumption and alcohol consumption, and separate models by age were formed (Additional file 3: Table S7). Infrequent energy drink consumption was associated with short sleep only among 13-year-olds as compared to 15-year-olds. Moreover, the increase in the frequency of energy drink consumption (from infrequent to frequent consumption) indicated a stronger association with short sleep and with alcohol consumption among 13-year-olds as compared to 15-year-olds.

#### Perceived unfavorable health indicators

As compared to non-consumers, frequent energy drink consumers were more likely to report low self-rated health, multiple health complaints and feelings of insufficient sleep, even after control for several salient covariates (Table 4).

**Table 4:** Perceived negative health indicators by energy drink consumption: adjusted odds ratios (aOR), 95% confidence intervals (CI), and corresponding relative risks (RR) and 95% confidence intervals (CI)^d^

		**Low self-rated health**^**a**^	**Multiple health complaints**^**b**^	**Feelings of insufficient sleep**^**a**^
Energy drinks				
No consumption		1.00	1.00	1.00
Infrequent consumption	aOR 95% CI *P*-value	1.20 [0.85–1.71] 0.299	1.15 [0.88–1.49] 0.306	0.97 [0.68–1.37] 0.846
	RR 95% CI	1.12 [0.87–1.55]	1.10 [0.91–1.29]	0.97 [0.70–1.32]
Frequent consumption	aOR 95% CI *P*-value	1.61 [1.18–2.21] 0.003	1.52 [1.11–2.09] 0.010	1.67 [1.12–2.48] 0.011
	RR 95% CI	1.48 [1.15–1.87]	1.31 [1.07–1.56]	1.56 [1.11–2.15]
Infrequent vs. Frequent^c^	*P*-value	0.251	0.166	0.010

Interaction was found in the models for low self-rated health and multiple health complaints, adjusted effect presented. The interaction effect presented in the Additional file 4: Table S8

^a^Models were adjusted for age, gender, family affluence, short sleep, low physical activity, current smoking, alcohol consumption, and multiple health complaints

^b^Model was adjusted for age, gender, family affluence, short sleep, low physical activity, current smoking, and alcohol consumption

^c^Tested with pairwise multiple comparisons

^d^Relative risks and their confidence intervals were derived from adjusted odds ratios

Interactions were found regarding low self-rated health and multiple health complaints, and separate models by age and/or gender were formed (Additional file 4: Table S8). Age moderated the association between energy drink consumption and low self-rated health, and between energy drink consumption and multiple health complaints. Frequent energy drink consumption was associated with an increased risk of reporting low self-rated health and multiple health complaints only among 13-year-olds as compared to 15-year-olds. As regards gender differences, both infrequent and frequent consumption of energy drinks were associated with an increased risk of reporting multiple health complaints only among girls.

## Discussion

Our results support the concerns highlighted in international scientific discussion regarding energy drink consumption among adolescents. Our findings add to the growing body of research clearly indicating that adolescents who consume energy drinks have a greater chance of reporting health-compromising behaviors and perceived negative health indicators than those who do not consume energy drinks. This was the case even among persons as young as 13 years, and after control for possible confounders was applied. Hence, evaluation of the appropriateness of energy drinks for adolescents should go beyond a focus on the direct physiological effects of energy drinks. Our study responds to the global need to obtain knowledge on energy drinks, in order to update existing policies and construct new ones. The aim would be to reduce energy drink consumption, bearing in mind that most countries have no legal restrictions on their sale. In some European countries (such as Finland and the UK) the government has proposed restrictions on energy drink sales to children aged under 16 [9, 33]; however, implementation has yet to occur. Latvia and Lithuania have banned the sale of energy drinks to persons under the age of 18 ([34], see also [35]).

One of the main outcomes of this study was that even infrequent consumption of energy drinks is associated with several negative health indicators, representing possible risk factors for adolescents’ health and well-being. Moreover, our results suggest that the reporting of several health-compromising behaviors significantly increased in parallel with energy drink consumption. While causal relationships cannot be established, the findings strengthen the evidence for a “frequency–response” relationship between the health-compromising behaviors assessed and energy drink consumption (e.g. [11, 15, 19]). The co-occurrence of energy drink consumption and negative health indicators is alarming, given that energy drink consumption *per se* has previously proven associations with adverse health effects among adolescents, due to their psychoactive ingredients, especially caffeine [36]. Moreover, considering the developmental period of adolescence, most of the health-compromising behaviors examined can themselves put adolescents at risk.

One exception in our results was the association between energy drink consumption and low physical activity. In contrast with other health indicators, we found no association between frequent energy drink consumption and low physical activity, and in fact infrequent energy drink consumers showed a lesser likelihood of reporting low physical activity than non-consumers. It can be suggested that – given our results and the lack of consensus in previous studies regarding the association between energy drink consumption and physical activity [14, 17, 18, 21] – this association requires further study.

One particularly concerning finding in our study was the clear associations between energy drink consumption and substance use, in line with previous studies [11–18], with evidence also on the use of snus – a phenomenon that has received only limited attention in the literature on energy drink consumption. Leal & Jackson. [37] found that differences in daily-level consumption explained intentions to use cannabis. In our study, even weekly (i.e. frequent) and less than weekly (infrequent) consumption of energy drinks was already linked to cannabis use, as compared to non-consumption of energy drinks. Given the psychoactive properties of the ingredients in energy drinks and in other harmful substances, their co-consumption is potentially harmful for adolescent health, and the associations would need more detailed examination using a range of methodologies and data. Adolescence is critical in establishing a foundation for health, and once they are initiated, health behaviors track into adulthood [2]. In fact, our results overall suggest that as compared to non-consumers, energy drink consumers have poorer adherence to health-enhancing behaviors. To take an example, the co-occurrence of energy drink consumption and inadequate tooth brushing is problematic for oral health because of the sugar content and high acidity of energy drinks; these increase the risk of dental problems such as dental caries and tooth erosion [38].

These associations are notably problematic because there is a contradiction between the reasons for consumption and the associated indicators. In consuming energy drinks, adolescents pursue improved functioning, involving stronger concentration [39] and increased energy levels [4]. These are in fact the effects on which the marketing of the drinks is based [40, 41]. However, as our results indicate, energy drink consumption is associated with short sleep and skipping breakfast, both of which may lead to poorer cognitive functioning [42].

Prominent changes related to sleep (e.g., timing and wakefulness) take place during adolescence [43]. Hence, the association between energy drink consumption and short sleep is a matter of concern, given that caffeine – which is the major constituent of energy drinks – acts as an antagonist of adenosine [6], which plays a key role in regulating the sleep-wake cycle. This may lead to a vicious circle such that adolescents have short sleep and feel tired in the morning. They then pursue increased energy from energy drinks and subsequently have problems in falling asleep, due to their caffeine intake. In the present study, in addition to short sleep, energy drink consumption was indeed associated with feelings of insufficient sleep, in line with previous studies [19]. This phenomenon poses a risk for learning and for mood regulation [43], and one can see that the consumption may have precisely the opposite effect from that originally sought. Consistent with these associations, we further discovered that consumption of energy drinks was associated with low self-rated health and with experiencing multiple health complaints, in line with previous studies [13, 19, 44]. In a review by Khouja et al. [9], it was suggested that energy drink consumption might either contribute to poorer health or vice-versa, or else that they might share a common cause. Recent research [45] has suggested that the associations between energy drink consumption and both emotional and behavioral problems are mediated by the amount of sleep and breakfast consumption. Thus, more research is needed on the associations between different health compromising behaviors and energy drinks, and on the possible mediating and moderating roles in these associations.

To prevent a vicious circle from forming, one can suggest that comprehensive interventions are needed to ensure that adolescents, parents/guardians, and professionals working with adolescents (e.g., in schools) should have adequate and reliable information on energy drinks, their individual effects, and their role in adolescents’ lives as a whole. It has previously been observed that adolescents have only limited knowledge on the constituents of energy drinks [40, 41]. Our own previous study [46] suggests that higher health literacy may act as a preventive factor, indicating the need for adolescents to gain competencies that will allow them to evaluate the claims made in the marketing of energy drinks. These claims are notably made in relation to everyday activities, such as studying, and they reach adolescents via social media platforms [47], within which leading energy drink brands are widely visible. One can suggest that the marketing of energy drinks in platforms popular among adolescents needs more rigorous evaluation. Interestingly, our results also indicate an association between energy drink consumption and problematic social media use – a phenomenon which itself calls for further examination.

Overall, the findings highlight the need for health promotion and health education measures targeted at adolescents’ energy drink consumption, with the aim of supporting adolescents’ competencies to maintain healthy daily rhythms and thus maintain their vitality in a sustainable way. The associations found in this study suggest that energy drink consumption may act as an indicator of the co-occurrence of other health-compromising behaviors among adolescents. In a recent longitudinal study [48], energy drink consumption was not only associated with health-compromising behaviors, but also a predictor of worse health one year later. Even though the precise causality of the associations regarding our study remains unclear and should be further studied, there are grounds for suggesting that a decrease in energy drink consumption could have positive effects on several health-compromising behaviors and their consequences.

The evidence of this study seems to support policy-level initiatives. In Finland, a current government program includes the aim of restricting energy drink sales to adolescents under 16 years old, but implementation has yet to occur. Other policy actions are also being considered in Finland. The government program includes the aim of promoting health via taxes; in particular, it has been proposed that the current tax on sugar-sweetened drinks should be raised.

### Strengths and limitations

The strengths of the study include a large and nationally representative sample, validated measures, and a comprehensive selection of indicators encompassing adolescents’ health behaviors and perceived health. Moreover, one of the strengths of the study is that it assesses how adolescents are affected by energy drinks at different levels of exposure (measured here in terms of frequency of use), with control for possible confounders. The present study sheds light on the moderating effect of age and gender on the associations between energy drink consumption and negative health indicators. A limitation of the study might be considered the self-reported nature of this study. The responses in this regard could be subject to bias arising from the social desirability, or from the recall period. It should also be noted that the survey does not include a question on the *amounts* of energy drinks consumed at a given time. Our results are nevertheless valuable in indicating that in studying the consumption of energy drinks one should pay attention to frequency, given that (as shown by our results) energy drink consumers do not constitute one homogenous group. Moreover, we addressed gaps in the literature by including health behaviors such as tooth brushing and the use of snus, which have received too little attention in the literature as regards their associations with energy drink consumption.

## Conclusions

Energy drink consumption, even infrequent, was associated with several negative health indicators. The reporting of several health-compromising behaviors increased with the frequency of energy drinks consumption. Comprehensive interventions and actions are needed to ensure that adolescents have adequate information on energy drinks, on their individual effects, and on their possible consequences, including their co-occurrence with other health-compromising behaviors. Parents/guardians and professionals working with adolescents should be aware of the possible risk for adolescent health and well-being caused by these associations, the aim being to support adolescents’ competencies to maintain healthy daily rhythms and adherence to healthy everyday habits. Policy-level actions such as restrictions on the sale of energy drinks to adolescents are needed, and the marketing of these beverages in platforms popular among adolescents (e.g., the social media) should be rigorously evaluated.

## Supplementary Information

**Additional file 1: Table S5. **Comparison of negative health indicators by energy drink consumption between 13- and 15-year-olds, Pearson's chi-squared test.

**Additional file 2: Table S6. **Prevalence of health-compromising behaviors and perceived negative health indicators among 13- and 15-year-olds.

**Additional file 3: Table S7.** Health-compromising behaviors by energy drink consumption, models with interactions: adjusted odds ratios (aOR), 95% confidence intervals (CI), and corresponding relative risks (RR) and 95% confidence intervals (CI).

**Additional file 4: Table S8.** Perceived negative health indicators by energy drink consumption, models with interactions: adjusted odds ratios (aOR), 95% confidence intervals (CI), and corresponding relative risks (RR) and 95% confidence intervals (CI).

## Data Availability

The HBSC Data Management Centre distributes data in accordance with the HBSC data access policy. Requests to access these datasets should be directed to dmc@hbsc.org.
