# Supplementary material for: Associations between adolescents’ energy drink consumption frequency and several negative health indicators
Source: BMC Public Health. 2023 Feb 6;23:258. doi: 10.1186/s12889-023-15055-6 (PMC9903583; doi:10.1186/s12889-023-15055-6)
Supplement: Supplementary file 1 — Additional file 1: Table S5. Comparison of negative health indicators by energy drink consumption between 13- and 15-year-olds, Pearson's chi-squared test. [file 12889_2023_15055_MOESM1_ESM.docx]

|  | **13- vs. 15-year-olds, *P*-values** | | |
| --- | --- | --- | --- |
|  | **Frequent**  **consumers** | **Infrequent**  **consumers** | **Non-consumers** |
| Inadequate tooth brushing | 0.217 | 0.090 | 0.245 |
| Skipping  breakfast | 0.083 | 0.859 | 0.057 |
| Low physical  activity | 0.005 | 0.001 | <0.001 |
| Short sleep | 0.740 | 0.126 | 0.003 |
| Problematic  social media use | 0.220 | 0.901 | 0.622 |
| Current  smoking | <0.001 | <0.001 | <0.001 |
| Alcohol  consumption | <0.001 | <0.001 | <0.001 |
| Low self-rated  health | 0.004 | 0.833 | 0.145 |
| Multiple health complaints | 0.080 | 0.107 | 0.069 |
| Feelings of  insufficient sleep | 0.906 | 0.199 | 0.038 |

Additional file 1: Table S5 Comparison of negative health indicators by energy drink consumption between 13- and 15-year-olds, Pearson's chi-squared test
