# Supplementary material for: Associations between adolescents’ energy drink consumption frequency and several negative health indicators
Source: BMC Public Health. 2023 Feb 6;23:258. doi: 10.1186/s12889-023-15055-6 (PMC9903583; doi:10.1186/s12889-023-15055-6)
Supplement: Supplementary file 2 — Additional file 2: Table S6. Prevalence of health-compromising behaviors and perceived negative health indicators among 13- and 15-year-olds. [file 12889_2023_15055_MOESM2_ESM.docx]

Additional file 2: Table S6 Prevalence of health-compromising behaviors and perceived negative health indicators among 13- and 15-year-olds.

|  | **13-year-olds** | **15-year-olds** | **Total** |
| --- | --- | --- | --- |
| Inadequate tooth brushing | 35.7  [32.3–39.2] | 36.6  [33.1–40.3] | 36.2  [33.7–38.7] |
| Skipping breakfast | 19.3  [16.6–22.3] | 25.5  [22.8–28.5] | 22.5  [20.5–24.6] |
| Low physical activity | 10.6  [8.6–13.0] | 21.2  [18.7–24.0] | 15.9  [14.1–18.0] |
| Short sleep | 19.1  [16.6–21.9] | 26.2  [23.2–29.4] | 22.7  [20.6–24.9] |
| Problematic social media use | 11.1  [8.8–14.0] | 11.2  [9.2–13.6] | 11.2  [9.6–12.9] |
| Current smoking | 5.3  [4.0–7.0] | 15.3  [12.9–18.2] | 10.4  [8.7–12.4] |
| Alcohol consumption | 13.7  [11.3–16.5] | 36.1  [31.5–41.0] | 24.9  [21.6–28.5] |
| Drunkenness |  | 25.3  [22.1–28.8] |  |
| Current snus use |  | 12.5  [10.5–14.8] |  |
| Cannabis use |  | 11.1  [9.2–13.4] |  |
| Low self-rated health | 19.4  [17.3–21.7] | 19.9  [17.2–22.8] | 19.6  [17.8–21.6] |
| Multiple health complaints | 33.8  [30.2–37.5] | 38.1  [35.2–41.0] | 36.0  [33.6–38.4] |
| Feelings of insufficient sleep | 12.5  [10.4–14.9] | 16.8  [14.5–19.2] | 14.6  [13.0–16.4] |
| *n* | (1118–1237) | (1042–1167) | (2160–2403) |
