# Supplementary material for: Associations between adolescents’ energy drink consumption frequency and several negative health indicators
Source: BMC Public Health. 2023 Feb 6;23:258. doi: 10.1186/s12889-023-15055-6 (PMC9903583; doi:10.1186/s12889-023-15055-6)
Supplement: Supplementary file 3 — Additional file 3: Table S7. Health-compromising behaviors by energy drink consumption, models with interactions: adjusted odds ratios (aOR), 95% confidence intervals (CI), and corresponding relative risks (RR) and 95% confidence intervals (CI). [file 12889_2023_15055_MOESM3_ESM.docx]

Additional file 3: Table S7 Health-compromising behaviors by energy drink consumption, models with interactions: adjusted odds ratios (aOR), 95% confidence intervals (CI), and corresponding relative risks (RR) and 95% confidence intervals (CI)^a^.

|  |  | **Short sleep** |  |  | **Alcohol consumption** | |
| --- | --- | --- | --- | --- | --- | --- |
|  |  | **13-year-olds^b^** | **15-year-olds^b^** |  | **13-year-**  **olds^c^** | **15-year-**  **olds^c^** |
| Energy drinks |  |  |  |  |  |  |
| No   consumption |  | 1.00 | 1.00 |  | 1.00 | 1.00 |
|  |  |  |  |  |  |  |
| Infrequent  consumption | aOR  95% CI P-value | 2.08  [1.37–3.16]  0.001 | 1.32  [0.94–1.85]  0.103 |  | 2.10  [1.21–3.65]  0.009 | 2.25  [1.52–3.31]  <0.001 |
|  | RR | 1.86 | 1.25 |  | 1.97 | 1.79 |
|  | 95% CI | [1.32–2.55] | [0.95–1.60] |  | [1.20–3.14] | [1.37–2.24] |
|  |  |  |  |  |  |  |
| Frequent  consumption | aOR  95% CI P-value | 4.51  [2.83–7.19]  <0.001 | 1.57  [1.03–2.39]  0.035 |  | 4.92  [2.91–8.30]  <0.001 | 3.27  [2.17–4.92]  <0.001 |
|  | RR | 3.25 | 1.42 |  | 3.97 | 2.23 |
|  | 95% CI | [2.35–4.26] | [1.02–1.90] |  | [2.61–5.74] | [1.75–2.72] |
|  |  |  |  |  |  |  |
| Infrequent vs.  frequent^d^ | P-value | 0.004 | 0.756 |  | 0.015 | 0.139 |

^a^ Relative risks and their confidence intervals were derived from adjusted odds ratios.
^b^ Adjusted for gender, family affluence, low physical activity, current smoking, alcohol consumption, and multiple health complaints.
^c^ Adjusted for gender, family affluence, short sleep, low physical activity, current smoking, and multiple health complaints.
^d^ Tested with pairwise multiple comparisons.
