# Supplementary material for: Associations between adolescents’ energy drink consumption frequency and several negative health indicators
Source: BMC Public Health. 2023 Feb 6;23:258. doi: 10.1186/s12889-023-15055-6 (PMC9903583; doi:10.1186/s12889-023-15055-6)
Supplement: Supplementary file 4 — Additional file 4: Table S8. Perceived negative health indicators by energy drink consumption, models with interactions: adjusted odds ratios (aOR), 95% confidence intervals (CI), and corresponding relative risks (RR) and 95% confidence intervals (CI). [file 12889_2023_15055_MOESM4_ESM.docx]

Additional file 4: Table S8 Perceived negative health indicators by energy drink consumption, models with interactions: adjusted odds ratios (aOR), 95% confidence intervals (CI), and corresponding relative risks (RR) and 95% confidence intervals (CI)^a^.

|  |  | **Low self-rated health** | |  | **Multiple health complaints** | | | |
| --- | --- | --- | --- | --- | --- | --- | --- | --- |
|  |  | **13-year-olds^b^** | **15-year-olds^b^** |  | **13-year-**  **olds^c^** | **15-year-**  **olds^c^** | **Girls^d^** | **Boys^d^** |
|  |  |  |  |  |  |  |  |  |
| Energy drinks |  |  |  |  |  |  |  |  |
| No   consumption |  | 1.00 | 1.00 |  | 1.00 | 1.00 | 1.00 | 1.00 |
|  |  |  |  |  |  |  |  |  |
| Infrequent  consumption | aOR  95% CI P-value | 1.33  [0.80–2.22]  0.273 | 1.14  [0.69–1.88]  0.602 |  | 1.29  [0.87–1.92]  0.208 | 1.18  [0.85–1.63]  0.326 | 1.62  [1.13–2.32]  0.010 | 0.83  [0.55–1.24]  0.354 |
|  | RR | 1.27 | 1.11 |  | 1.19 | 1.11 | 1.31 | 0.86 |
|  | 95% CI | [0.82–1-91] | [0.73–1.64] |  | [0.90–1.52] | [0.90–1.34] | [1.08–1.55] | [0.61–1.18] |
|  |  |  |  |  |  |  |  |  |
| Frequent  consumption | aOR  95% CI P-value | 2.00  [1.24–3.22]  0.005 | 1.30  [0.84–1.99]  0.237 |  | 1.90  [1.22–2.96]  0.005 | 1.19  [0.78–1.79]  0.416 | 1.81  [1.17–2.81]  0.008 | 1.24  [0.84–1.85]  0.280 |
|  | RR | 1.77 | 1.24 |  | 1.52 | 1.11 | 1.39 | 1.18 |
|  | 95% CI | [1.20–2.49] | [0.86–1.71] |  | [1.15–1.91] | [0.84–1.41] | [1.10–1.67] | [0.87–1.57] |
|  |  |  |  |  |  |  |  |  |
| Infrequent vs.  frequent^e^ | P-value | 0.181 | 0.947 |  | 0.244 | 1.000 | 0.934 | 0.107 |

^a^ Relative risks and their confidence intervals were derived from adjusted odds ratios.
^b^ Adjusted for gender, family affluence, low physical activity, short sleep, current smoking, alcohol consumption, and multiple health complaints.
^c^  Adjusted for gender, family affluence, low physical activity, short sleep, current smoking, and alcohol consumption.
^d^ Adjusted for age, family affluence, low physical activity, short sleep, current smoking, and alcohol consumption. ^e^ Tested with pairwise multiple comparisons.
